# Draft Genome Sequence of Paraburkholderia sp. Strain C35, Isolated from a Malaysian Tropical Peat Swamp Forest

**DOI:** 10.1128/genomeA.00561-18

**Published:** 2018-06-21

**Authors:** Chin Chin Too, Kuan Shion Ong, Markus J. Ankenbrand, Sui Mae Lee, Catherine M. Yule, Alexander Keller

**Affiliations:** a School of Science, Monash University Malaysia, Petaling Jaya, Malaysia; b Tropical Medicine & Biology Multidisciplinary Platform, Monash University Malaysia, Petaling Jaya, Malaysia; c Department of Bioinformatics, Biocenter, University of Würzburg, Am Hubland, Würzburg, Germany; d Center for Computational and Theoretical Biology, University of Würzburg, Am Hubland, Würzburg, Germany

## Abstract

We report the draft genome sequence of a bacterial isolate, Paraburkholderia sp. strain C35, which was isolated from a Malaysian tropical peat swamp forest. The putative genes for the biogeochemical processes were annotated and are publicly available in the online databases.

## GENOME ANNOUNCEMENT

Tropical peat swamp forests (TPSF) are extreme ecosystems, most commonly found in Southeast Asia, especially in Indonesia and Malaysia. They play an important role as terrestrial carbon reservoirs, as they sequester carbon in their substrate of semi-decomposed plant debris known as peat. TPSF harbor high microbial diversity (1), and the microbial communities have been documented to produce enzymes involved in plant biomass degradation (2) and also antimicrobial compounds (3, 4).

Bacterial isolation was conducted using peat samples collected from North Selangor Peat Swamp Forest, Malaysia, in order to investigate putative functional genes involved in carbon and nitrogen cycles. The bacterium Paraburkholderia sp. strain C35 was isolated at Monash University Malaysia, using surface peat sampled in December 2016. The isolate was cultured on minimal medium using lignin as the carbon source (5), after enrichment on primary enrichment medium (6). DNA extraction was performed from the culture incubated in tryptic soy broth for 36 h at 30°C, according to the protocol of the QIAamp DNA Mini Kit (Qiagen, Hilden, Germany). Library preparation was conducted based on the protocol of the Nextera XT DNA library preparation kit (Illumina, San Diego, CA), and the whole-genome sequencing was performed on the Illumina NextSeq 500 (150-bp paired-end reads) at the Biocenter, University of Würzburg, Germany. The raw reads were corrected and assembled *de novo* using SPAdes v3.10.1 (7), followed by gene annotation in Prokka v1.12 (8). The Enzyme Commission (EC) numbers were used to construct the metabolic pathways on the Kyoto Encyclopedia of Genes and Genomes (KEGG) database (9). In addition, the amino acid file was uploaded to BlastKOALA (10) for gene annotation and pathway mapping on the KEGG database.

The genome contained 224 contigs with a total length of 9,705,730 bp (*N*_50_, 69,876 bp) and 61.49% GC content. There are 8,807 coding DNA sequences (CDSs), 1 transfer-messenger RNA (tmRNA), 7 rRNAs, and 59 tRNAs found within the genome. Functional genes encoding enzymes such as methanol dehydrogenase, glutathione-independent formaldehyde dehydrogenase, formate dehydrogenase, *S*-formylglutathione hydrolase, and *S*-(hydroxymethyl) glutathione dehydrogenase were detected, indicating that Paraburkholderia sp. strain C35 was potentially able to consume methanol to form formaldehyde and formate and finally produce CO_2_. This strain was also predicted to perform cellulose and pectin degradation with the presence of various hydrolase genes such as alpha-amylase, cellulase, pectinase, and beta-glucosidase. It showed a potential role in the nitrogen cycle, because it possessed a nitrite reductase gene, which is associated with nitrite reduction to ammonium in the second step of dissimilatory nitrate reduction. Further investigation of the genome will generate more insights on the potential roles this strain plays in the acidic and extreme tropical peat swamp forest ecosystems.

### Accession number(s).

This whole-genome shotgun project has been deposited at DDBJ/ENA/GenBank under the accession number QBUY00000000. The version described in this paper is version QBUY01000000.
